# 

**Published:** 1898-01

**Authors:** 


### INDEX

TO THE

### HOMŒOPATHIC PHYSICIAN.

### VOLUME XVIII, 1898.

PAGE

[illegible]omen, Gunshot Wound of, with Twelve Perforations. W. F. Cole ... 482
[illegible]ed Therapy, An. Dr. Schuess[illegible]er: Review of ... 554
[illegible]t, A Remarkable. J. Fitz[illegible]hew ... 135
Aceta [illegible] Thallium ... 465
Actea [illegible]emosa. C. L. Olds ... 291
African Colonial Enterprise, Review of 277
After Fish Eat Cheese. B. Fincke ... 269
Alaska: Its Neglected Past; Its Brilliant Future. Review of ... 417
American Medical Publishers' Association ... 140
Antidotal Method. B. Fincke ... 133
Anti-vaccination Nuts; Reply to Dr. Orme's. W. B. Clarke ... 359
Appendicitis and Money ... 520
Arsenic: Crew got fat on ... 559
Arizona: Climatography of Salt River Region of. Review of ... 517
Augur, Geo. J. Syphilis and Sycosis. 159

Back, Repertory of. E. H. Wilsey...20, 68, 110, 148, 191, 249, 306, 406, 441, 484
Bacteria, Province of. B. Fincke ... 222
Barometrical Pressure as a Factor in Medicine. T. Wesley Burwood ... 471
Bell, Dr J. S.—Removal ... 325
Betts, Dr. B. F., Recovery of ... 519
Biochemic Treatment: Review of ... 554
Book Notices ... 48, 92, 177, 227, 276, 417, 463, 515, 554
Brasol, Leon. Hahnemann's Tomb ... 176
Burns, Treatment of ... 519
Butter Test ... 453
Burwood, T. Wesley. Barometrical Pressure as a Factor in Medicine 471

Calcarea, Perspiration of. J. M. Selfridge ... 373
Calculus Nasal. W. H. Poole ... 456
Campbell, Dr Alice B.—Removal ... 140
Campbell. J. B. Phoshorus a Peculiar Symptom ... 272
Cancer, Etiology of. W. B. Clarke ... 246

PAGE

Can Climatic Susceptibility be Cured? B. Fincke ... 39
Can Climatic Susceptibility be Cured? E. Carleton ... 40
Can Syphilis be Cured with High Potencies? J. M. Selfridge ... 281
Carleton, E. Can Climatic Susceptibility be Cured? ... 40
Cephalalgia. Dr. Wassily ... 396
Chemical Point of View, High Dilutions and their Effects from a. J. E. Gilpin ... 232
Children's Homœopathic Hospital of Philadelphia ... 140
Clarke, Wm. B. Etiology of Cancer ... 246
Reply to Dr. Orme's Anti-vaccination Nuts ... 359
Vaccination Causes Consumption, 509
Vaccination in the Army ... 462
Vaccination Papers Ought to be Welcomed ... 276
Vaccination Viewed by a Potent Enemy of Vaccination ... 324
Climatography of the Salt River Region of Arizona, Review of ... 517
Cole, W. Fisher. Gunshot Wound of Abdomen, with Twelve Perforations ... 482
Colocynth: A Lecture. C. L. Olds ... 35
Commentaries on *The Organon*. B. Fincke ... 342
Constipation in Babies ... 459
Consumption, Vaccination Causes. W. B Clarke ... 509
Cornea. Disease of. Dr. Wassily ... 397
Correction ... 324
Corrections in the February Number ... 158
*Critique, The.* Notice of ... 177
Crutcher, Howard. Phosphorus in Peritonitis ... 83
Cuba, Map of ... 177
Cushing, A. M. Response to the Toast History of Microbes ... 512

Diarrhœa, Mortality from ... 514
Dillingham, T. M. Vaccination Papers Approved ... 273

PAGE
Directory, Homœopathic — British, Colonial, and Continental. Review of.......... 518
Discrimination Against Homœopathists.......... 460
Diseases of the Skin, Review of.......... 463
Dobson, F. Vaccination Papers of Practical and Immediate Value, 274

Eclectic College Confederation.......... 465
Editorials .....1, 49, 93, 141, 181, 229, 273, 279, 419, 467, 521
Editorial. Notice to the Profession..... 141
Pulsatilla Pratensis..........419, 467, 521
Silicea.. ........ 1, 49, 93, 141, 181, 229, 279
Vaccination in the Light of the Royal British Commission......... 95
Vaccination Papers of Dr. Leverson.......... 273
Elements of Latin, Review of.......... 276
Epitome of the History of Medicine, Review of.......... 179
Etiology of Cancer. W. B. Clarke...... 246

Fincke, B. After Fish Eat Cheese...... 269
Antidotal Method.......... 133
Can Climatic Susceptibility be Cured ?.......... 39
Commentaries on *The Organon*...... 342
Province of Bacteria.......... 222
Surgery, Medicine, and a Little Logic.......... 422
Vaccination an Unhomœopathic Measure.......... 87
Vaccination Papers Should be in Every Number.......... 274
Fish, Fresh, Test of.......... 454
Fitz-mathew, J. A Remarkable Accident.......... 135
Typhlitis—Lachesis 2c.......... 226
Food Tests, Some Simple. E. A. Martin. 452
For Sale.......... 325
Fowler, S. Mills The Vaccination Papers a Waste of Time and Space.......... 372

Gage, L. J. The New War Loan.......... 320
Gastric Disorder. Dr. Wassily......399, 400
Gastric and Intestinal Disorders, Rational Treatment of. Review of.. 277
Germ Theory, an Inquiry into the Logical Basis of the. M. R. Leverson.......... 263
Gilpin, J. Elliott. High Dilutions and their Effects from a Chemical Point of View.......... 232
Gleet. Dr. Wassily.......... 401
Gunshot Wound of Abdomen, with Twelve Perforations, Recovery. W. F. Cole.......... 482

Hahnemann Medical College of Phila. Annual Reunion Alumni Association.......... 140
Hahnemann, Samuel. Psora.......... 173
Venereal Diseases.......... 163
Hahnemann's Cautions. G. E. Manning.......... 207
Hahnemann, Exhumation of the Body of, Official Account of.......... 327
Hahnemann, Monument to.......... 325
Hahnemann, Monument over Grave. B. W. James.......... 460
Hahnemann's Tomb. Leon Brasol. 176, 460

PAGE
Hahnemann's Tomb, International Commission for the Restoration of.......... 174
Haviland, N. H. Homing Pigeons as Medical Messengers.......... 316
Hawking Mucus, Vomiting from. A. B. Eadie.......... 86
Hearts, Two in One Man.......... 545
Hemorrhoids, Fistula, etc., Office Treatment of, Review of.......... 464
High Dilutions and their Effects from a Chemical Point of View J. Elliott Gilpin.......... 232
Homing Pigeons as Medical Messengers. N. H. Haviland.......... 316
Homœopathic Philosophy : Bureau of, 525
Homœopathists, Discrimination Against.......... 460
Homœopathy and Thackeray's Daughter.......... 520
Hotel Lithia.......... 325
Hygiene, Psychological. F. O. Pease... 525
Hygiene, Rural, Outlines of, Review of.......... 92

Impotence. Dr. Wassily.......... 401
In Memoriam—Dr. Wm. Pepper.......... 413
In Memoriam—Dr. J. Heber Smith..... 551
Inquiry into the Logical Basis of the Germ Theory. M. R. Leverson.. 263
*International Annual and Practitioners' Index for 1898*, Review of.......... 177
International Commission for the Restoration of Hahnemann's Tomb. 174
International Hahnemannian Association.......... 374
*International Medical Annual*, for 1898.......... 48

James, B. W. International Commission for the Restoration of Hahnemann's Tomb.......... 174
Monument over Hahnemann's Grave.......... 460
James, Walter M. See " Editorials "...
Jaundice : A Case of. J. M. Selfridge.. 261
Johnson, Charles E The Vaccination Papers Good Missionary Literature.......... 372
Jones's Picnic, Dr. Review of.......... 227

Kraft. Dr. Frank, and the Cleveland College.......... 549

Lachesis in Typhlitis. J. Fitzmathew.......... 226
Lachrymal Sac, Disease of. Dr. Wassily.......... 397
Latin, Elements of, Review of.......... 276
Leverson, M. R. An Inquiry into the Logical Basis of the Germ Theory.......... 263
Note to Cory's Testimony.......... 374
Pasteurism.......... 370
Reliability of Vaccination Testimony.......... 371
Vaccination in the Light of the Royal British Commission.......... 3, 53, 96, 144, 184, 383, 435, 501, 546
Vaccination in the Army.......... 461
Leverson, Dr. Vaccination Papers of .. 273
Leverson, M. R. Vaccination Testimony in the April No.......... 239
Loan : The New War. L. J. Gage....... 320
Longport, New Jersey.......... 374

PAGE
Lutze, F. H. Phosphorus—A Peculiar Symptom .... 270

MacCracken, S. G. Removed .... 418
Manning, Guy E. Hahnemann's Cautions .... 207
Manning, Dr. Guy E. Removal of .... 519
Marchand's Hydrozone .... 559
Martin, E. A. Some Simple Food Tests .... 452
Martin, Eleanor F. Psora as Taught by Hahnemann in the *Chronic Diseases* .... 166
McNeil, A. Translation of Natrum-muriaticum by Dr. Wassily .... 395
Medicine, History of, Review of .... 179
Medico-Chirurgical Society of Central New York .... 304
Mental Depression. By Dr. Wassily .. 395
Michigan Monthly Bulletin of Vital Statistics, Review of .... 515
Microbes, History of. Response to the Toast. A. M. Cushing .... 512
Milk Tests .... 455
*Mining Bulletin*, Notice of .... 177
Monument to Hahnemann .... 325
Monument over Hahnemann's Grave. B. W. James .... 460
Mucus, Hawking, Vomiting from. .... 86

Nasal Catarrh. Dr. Wassily .... 398
Natrum-muriaticum. By Dr. Wassily 395
Nervous Diseases with Homœopathic Treatment, Review of .... 515
New York Homœopathic Union .... 39
New York Post-Graduate Medical School .... 465
Noah After the Flood .... 466
Notes and Notices .... 48, 140, 325, 374, 418, 465, 559
Notice to the Profession, Editorial .... 141

Oakes, C. H. Vaccination Papers Should be Continued .... 273
Official Account of the Exhumation of the Body of Hahnemann .... 327
Olds, C. M. Actea-racemosa .... 291
Olds, C. L. Colocynth—A Lecture .... 35
*Organon*, Commentaries on. B. Fincke .... 342
*Organon* and Materia Medica Club of the Bay Cities of California .... 159, 164, 204, 258, 260, 296
Organotherapy, Rampant .... 245
Orme's Anti-vaccination Nuts, Dr. Reply to. W. B. Clarke .... 359
Outlines of Rural Hygiene. By H. B. Bashore. Review of .... 92

Pasteurism. M. R. Leverson .... 370
Pease, F. O. Psychological Hygiene ... 525
Pepper, Dr. William, In Memoriam .... 413
Peritonitis, Phosphorus in. Howard Crutcher .... 83
Perspiration of Calcarea. J. M. Selfridge .... 373
Phosphorus: A Peculiar Symptom. J. B. Campbell .... 272
Phosphorus: A Peculiar Symptom. F. H. Lutze .... 270
Phosphorus in Peritonitis. Howard Crutcher .... 83
Physician's Visiting List: Review of ... 558
Pneumonia with Hyperpyrexia. E. V. Ross .... 137

PAGE
Poem by the Editor .... 520
Poole, W. H. Rhinolith or Nasal Calculus .... 456
Portuguese Men .... 466
Practical Urinalysis and Urinary Diagnosis: Review of .... 558
Province of Bacteria. B. Fincke .... 222
Psora as Taught by Hahnemann in the *Chronic Diseases*. E. F. Martin .... 166
Psora. Hahnemann .... 173
Psora. J. M. Selfridge .... 299
Psychological Hygiene .... 525
Pulsatilla. Editorial .... 419, 467, 521
Pulte Medical College .... 466
Pyrogen in Typhoid. F. E. Watts .... 85

Rational Treatment of Gastric and Intestinal Disorders. Review of ... 277
Red Cross Society .... 519
Reliability of Leverson's Vaccination Testimony. M. R. Leverson .... 371
Repertory of the Back. E. H. Wilsey. 20, 68, 110, 148, 191, 249, 306, 406, 441, 484
Reply to Dr. Orme's Anti-vaccination Nuts. W. B. Clarke .... 359
Response to the Toast, "History of Microbes." A. M. Cushing .... 512
Reward, A Great .... 326
Rheumatism of Joints. Dr. Wassily ... 400
Rhinolith or Nasal Calculus. W. H. Poole .... 456
Ross. E. V. Pneumonia with Hyperpyrexia .... 137

Sanitarium, Brookside .... 559
Sanitarium, Dr. Givens's .... 374
Saw Palmetto; Its History, Botany, etc. Review of .... 518
Sawyer, E. W. Vaccination Papers Needed .... 275
Schuessler: Biochemic Treatment: Review of .... 544
*Scientific American* Special Navy Supplement. Review of .... 228
Selfridge, J. M. A Case of Jaundice ... 261
The Perspiration of Calcarea .... 373
Psora .... 299
Can Syphilis be Cured with High Potencies? .... 281
Wyethia Helenioides with Additional Provings .... 375
Silicea. Editorial..1, 49, 93, 141, 181, 229, 279
Skin, Diseases of .... 463
Smith, Dr. J. Heber, In Memoriam .... 551
Society of Homœopathicians .... 525
Storer, John—Removal .... 466
*Suggestive Therapeutics, Journal of*. Review of .... 519
Surgery in the Middle Ages .... 560
Surgery, Medicine, and a Little Logic. B. Fincke .... 422
Syphilis Cured with High Potencies, Can. J. M. Selfridge .... 281
Syphilis and Sycosis. G J. Augur .... 159

Thackeray's Daughter and Homœopathy .... 520
Thallium, Acetate of .... 465
Tebbs, Mr., Contribution to the Vaccination Testimony- .... 370, 383, 435, 501, 546
Typhlitis—Lachesis 2c. J. Fitz-Mathew 226
Typhoid, Pyrogen in. F. E. Watts .... 85

PAGE

Umbilical Cord, Treatment of............ 434
Urinalysis, and Urinary Diagnosis. C. W. Purdy: Review of............... 558

Vaccination in the Light of the Royal British Commission. M. R. Leverson.. 3, 53, 96, 144, 184, 383, 435, 501, 546
Vaccination in the Light of the Royal British Commission, Editorial... 95
Vaccination in the Army. M. R. Leverson ........................................ 461
Vaccination in the Army. W. B. Clarke........................................ 462
Vaccination Articles, Encouraged to Complete the. A. Wheeler........ 323
Vaccination Causes Consumption. W. B. Clarke.............................. 509
Vaccination Papers of Dr. Leverson. Editorial ........................................ 273
Vaccination Papers Approved. T. M. Dillingham.................................. 273
Vaccination Papers Good Missionary Literature. Chas. E. Johnson ........................................ 372
Vaccination Papers Needed. E. W. Sawyer........................................ 275
Vaccination Papers of Practical and Immediate Value. F. Dobson... 274
Vaccination Papers Should be Continued. C. H. Oakes.............. ... 273
Vaccination Papers Should be in Every Number. B. Fincke....... 274
Vaccination Papers Ought to be Welcomed. W. B. Clarke................ 276
Vaccination Papers a Waste of Time and Space. S. M. Fowler......... 372

PAGE

Vaccination Testimony in the Aprii No. M. R. Leverson................. 239
Vaccination Testimony, Reliability of Leverson's. M. R. Leverson...... 371
Vaccination Testimony Mr. Tebb's Contributions to........................ 370
Vaccination an Unhomœopathic Measure. B. Fincke...................... 87
Vaccination Viewed by a Potent Enemy of Vaccination. W. B. Clarke.................................... 324
Venereal Diseases................................ 163
Visiting List, Lindsay & Blakiston's.... 558
Vomiting from Hawking Mucus. A. B. Eadie.................................... 86

Wassily, Dr. Cephalalgia........................ 396
Chronic Nasal Catarrh............... 398
Disease of the Cornea.................... 397
Disease of the Lachrymal Sac... .... 397
Gastric Disorder.......................399, 400
Gleet ..... ........................... ......... 401
Impotence.......................................... 401
Mental Depression.......................... 395
Natrum-muriaticum ........................ 395
Rheumatism of Joints..................... 400
Watts, F. E. Pyrogen in Typhoid...... 85
Weak and Aged, for the........................ 466
Weather, Changeable, Valuable in..... 520
Wheeler, A. Encouraged to Complete the Vaccination Articles............. 323
Wilsey, E. H. Repertory of the Back. 20, 68, 110, 148, 191, 249, 306, 406, 441, 484
Wine, Speer's Port Grape.,................... 559
Wyethia Helenioides. With Additional Provings. J. M. Selfridge.... ........................................ 375

VOL. XVIII. JANUARY, 1898. No. 1.

LIBRARY
SURGEON GENERAL'S OFFICE
MAR.-7--1898

### THE
### HOMŒOPATHIC PHYSICIAN,

### A Monthly Journal of Homœopathic Materia Medica and Clinical Medicine.

51

*"He is a freeman whom truth makes free, and all are slaves beside."*
*"Seek the Truth: come whence it may, cost what it will."*

EDITED AND PUBLISHED BY

WALTER M. JAMES, M. D.

NOTICE.

***All articles for publication, books for review, business communications, etc., must be sent to Editor, 1231 Locust Street, Philadelphia.***

***Subscribers will please notice that this Journal will be sent until arrears are paid and it is ordered discontinued.***

SURGEON GEN'L'S OFFICE
LIBRARY

PHILADELPHIA:

